# Cryo-EM structure of the human CST–Polα/primase complex in a recruitment state

**DOI:** 10.1038/s41594-022-00766-y

**Published:** 2022-05-16

**Authors:** Sarah W. Cai, John C. Zinder, Vladimir Svetlov, Martin W. Bush, Evgeny Nudler, Thomas Walz, Titia de Lange

**Affiliations:** 1 Laboratory of Cell Biology and Genetics, The Rockefeller University, New York, NY USA; 2 Laboratory of Molecular Electron Microscopy, The Rockefeller University, New York, NY USA; 3 Department of Biochemistry and Molecular Pharmacology, New York University Grossman School of Medicine, New York, NY USA; 4 Howard Hughes Medical Institute, Chevy Chase, MD USA

**Keywords:** Electron microscopy, Cell biology

## Abstract

The CST–Polα/primase complex is essential for telomere maintenance and functions to counteract resection at double-strand breaks. We report a 4.6-Å resolution cryo-EM structure of human CST–Polα/primase, captured prior to catalysis in a recruitment state stabilized by chemical cross-linking. Our structure reveals an evolutionarily conserved interaction between the C-terminal domain of the catalytic POLA1 subunit and an N-terminal expansion in metazoan CTC1. Cross-linking mass spectrometry and negative-stain EM analysis provide insight into CST binding by the flexible POLA1 N-terminus. Finally, Coats plus syndrome disease mutations previously characterized to disrupt formation of the CST–Polα/primase complex map to protein–protein interfaces observed in the recruitment state. Together, our results shed light on the architecture and stoichiometry of the metazoan fill-in machinery.

## Abstract (web summary)

Cryo-EM analysis of the human CST–Polα/primase complex reveals a metazoan-specific mode of interaction between CST and DNA polymerase α that is proposed to function in telomeric recruitment of Polα/primase for C-strand maintenance.

## Main

**Fig. 1:**
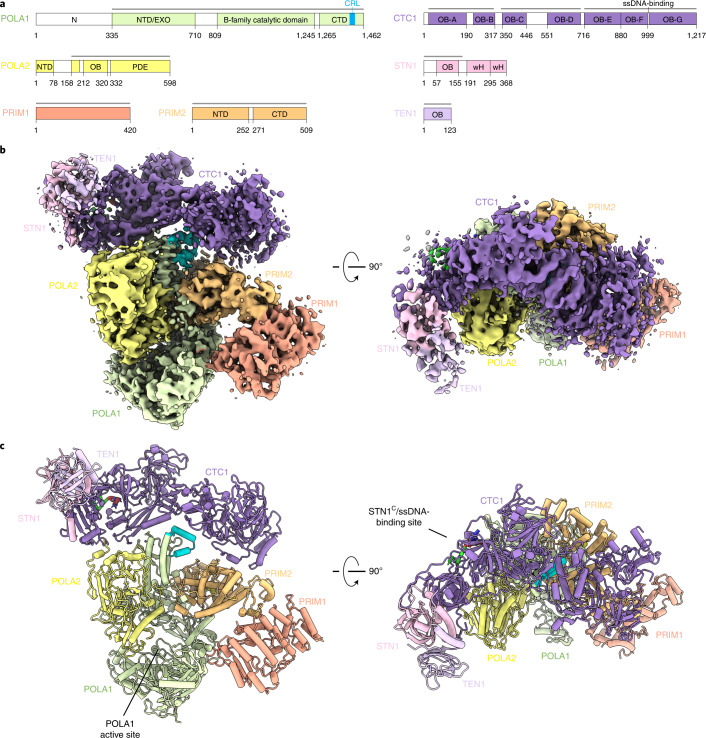
Architecture of the human CST–Polα/primase complex. **a**, Domain schematics for proteins used in this study. Gray bars indicate regions modeled in **c**. NTD, N-terminal domain; CTD, C-terminal domain; OB, oligonucleotide/oligosaccharide binding fold; PDE, phosphodiesterase domain; wH, winged helix-turn-helix motif. **b**, Orthogonal views of the cryo-EM map of CST–PP^ΔN^, segmented and colored by subunit as in **a**. **c**, Model of CST–PP^ΔN^ shown in cartoon representation.

Human telomeric DNA terminates in a 3′ overhang of the G-rich strand, which is required for t-loop formation and telomere protection^1^. The mature 3′ overhang must be regenerated during each cell cycle in a controlled manner. Following replication, nucleolytic resection of the 5′ strand can result in excessively long overhangs. The loss of sequences from the 5′ strand is counteracted through fill-in DNA synthesis by the CTC1–STN1–TEN1 (CST) complex and DNA polymerase α–primase (Polα/primase) (Fig. 1a), which are recruited to telomeres by the shelterin complex^2–7^. Dysfunctional fill-in, primarily driven by mutations in CST, causes the severe developmental disorder Coats plus syndrome (CP)^8,9^.

In addition to its telomeric function, CST–Polα/primase performs an analogous fill-in reaction at resected double-strand breaks (DSBs). At DSBs, CST–Polα/primase is recruited by the 53BP1–RIF1–shieldin complex through a direct interaction between CST and shieldin. The fill-in reaction executed by CST–Polα/primase at DSBs is a main determinant of the lethality of PARP inhibitors in BRCA1-deficient cells^10–12^. Although a structure of decameric CST bound to a short oligonucleotide has been determined^13^, the molecular details of how CST interacts with Polα/primase are largely unknown^14,15^. Here, we present a combination of structural, biochemical, and biophysical data describing the molecular basis of their interaction. Our structure of the complex in a recruitment state reveals a novel interface between Polα/primase and CST that evolved in metazoans and informs on CP mutations found in the N-terminal oligonucleotide/oligosaccharide-binding (OB) folds of CTC1.

## Results

### Architecture of CST–Polα/primase in a pre-catalysis state

**Extended Data Fig. 1:**
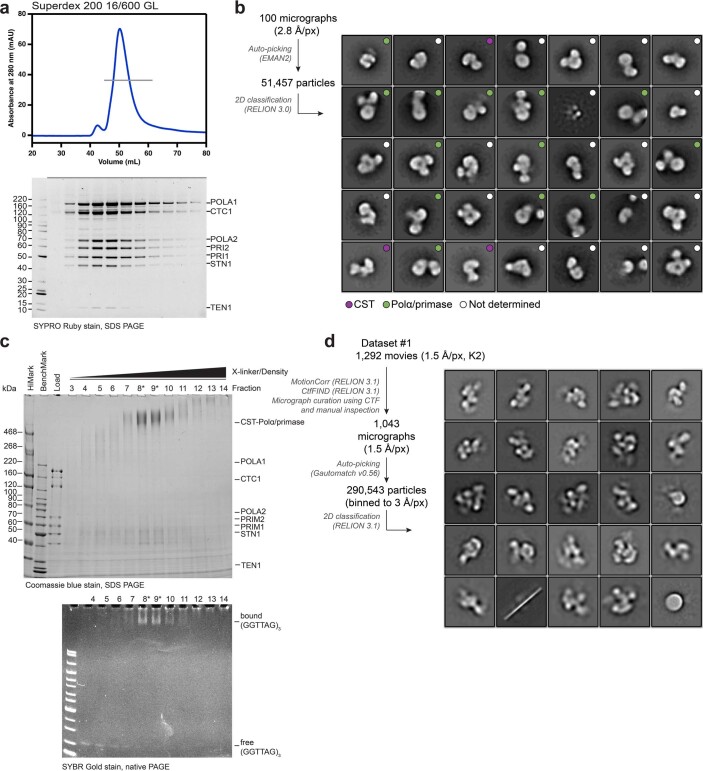
Reconstitutions of CST–PP^FL^. **a**, SEC-elution profile of CST–PP^FL^ (top). The grey line indicates the fractions visualized on the SYPRO Ruby-stained SDS-PAGE gel (bottom). This reconstitution was independently reproduced at least three times with similar results. **b**, Negative-stain EM image-processing pipeline and averages of the 35 (of 50) most populated 2D classes of the reconstituted complex characterized in **a**. The size of the 128-px box corresponds to 358 Å. **c**, GraFix preparation of CST–PP^FL^–ssDNA. *Top:* Coomassie blue-stained SDS-PAGE gel (4–12% (w/v) Tris-Glycine; Invitrogen) of GraFix fractions, showing formation of cross-linked CST–PP^FL^ species. Fractions marked with an asterisk (*) were pooled for analysis by cryo-EM. *Bottom:* SYBR Gold-stained native PAGE gel (4–20% (w/v) TBE; Invitrogen) of GraFix fractions, showing free and bound ssDNA. This reconstitution was independently reproduced at least three times with similar results. **d**, Cryo-EM image-processing pipeline and 2D-class averages obtained after vitrification and imaging of the GraFixed complex shown in **c**. See Table 1 for full data collection and processing details. Source data

**Extended Data Fig. 2:**
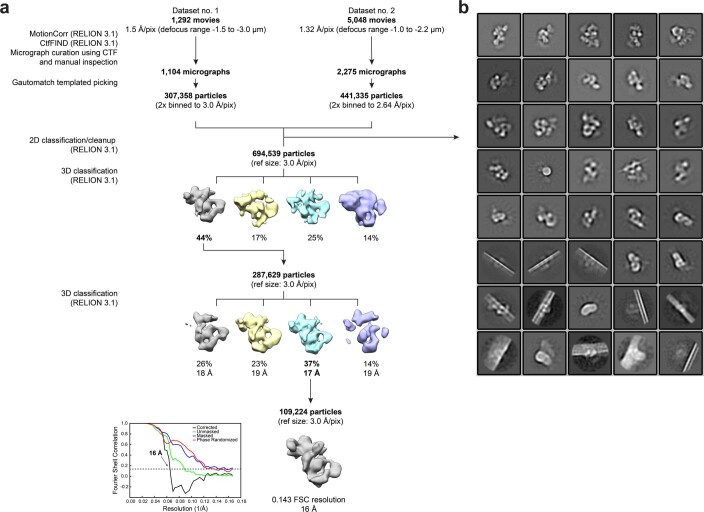
Cryo-EM image-processing pipeline of CST–PP^FL^. **a**, Cryo-EM image-processing pipeline used for the CST–PP^FL^ complex. Two datasets were combined in *RELION 3.1* as separate optics groups, and particles were refined together with a reference generated from dataset no. 1. See Table 1 for full data collection and processing details. **b**, 2D-class averages from a cleanup step prior to 3D classification. Classes clearly representing ice contamination or graphene oxide edges were removed during cleanup.

We sought to structurally characterize the complex using recombinant human CST and Polα/primase purified from insect cells. Although we could reconstitute a stable complex of CST–Polα/primase using size-exclusion chromatography (SEC), negative-stain EM images showed dissociation of CST and Polα/primase (Extended Data Fig. 1a,b). We added a (GGTTAG)_3_ substrate, as telomeric single-stranded DNA (ssDNA) has previously been shown to stabilize CST^13^, and used GraFix^16^ to cross-link the complex, resulting in a higher proportion of intact complexes in cryo-EM two-dimensional (2D) averages from a small dataset (no. 1; Extended Data Fig. 1c,d and Table 1). We collected an additional dataset from the same grid (no. 2) and could unambiguously identify CST and Polα/primase in the three-dimensional (3D) reconstructions. Although the mode of interaction between the two complexes appeared to be conserved in all maps, substantial conformational heterogeneity in peripheral regions limited the resolution to >16 Å (Extended Data Fig. 2 and Table 1).

**Table 1:** Cryo-EM data collection, refinement and validation statistics

	CST–PP^FL^ (dataset 1) EMD-26347	CST–PP^FL^ (dataset 2) EMD-26347	CST–PP^ΔN^ EMD-26346 PDB 7U5C
**Data collection and processing**			
Magnification	×28,000	×53,000	×64,000
Voltage (kV)	200	300	300
Electron exposure (e–/Å^2^)	53	57	52
Defocus range (μm)	−1.5 to −3	−1 to −2.2	−1 to −2.2
Pixel size (Å)	1.5	1.32	1.08
Symmetry imposed	*C*_1_	*C*_1_	*C*_1_
Initial particle images (no.)	307,358	441,335	2,515,853
Final particle images (no.)	109,224 (with no. 2)	109,224 (with no. 1)	131,850
Map resolution (Å)	16	16	4.6
FSC threshold	0.143	0.143	0.143
Map resolution range (Å)	Not calculated	Not calculated	4.0–8.0
**Refinement**			
Initial model used (PDB code)	PDB 5EXR PDB 6W6W	PDB 5EXR PDB 6W6W	PDB 5EXR PDB 6W6W
Model resolution (Å)	N/A	N/A	4.7
FSC threshold			0.5
Model resolution range (Å)	N/A	N/A	4–8
Map sharpening *B* factor (Å^2^)	–311	–311	–193
Model composition	N/A	N/A	
Non-hydrogen atoms			30,077
Protein residues			3740
Ligands			5
*B* factors (Å^2^)	N/A	N/A	
Protein			103.2
Ligand			110.7
R.m.s. deviations	N/A	N/A	
Bond lengths (Å)			0.006
Bond angles (°)			0.849
Validation	N/A	N/A	
MolProbity score			2.60
Clashscore			27.01
Poor rotamers (%)			0.12
Ramachandran plot	N/A	N/A	
Favored (%)			83.69
Allowed (%)			16.31
Disallowed (%)			0.00

**Extended Data Fig. 3:**
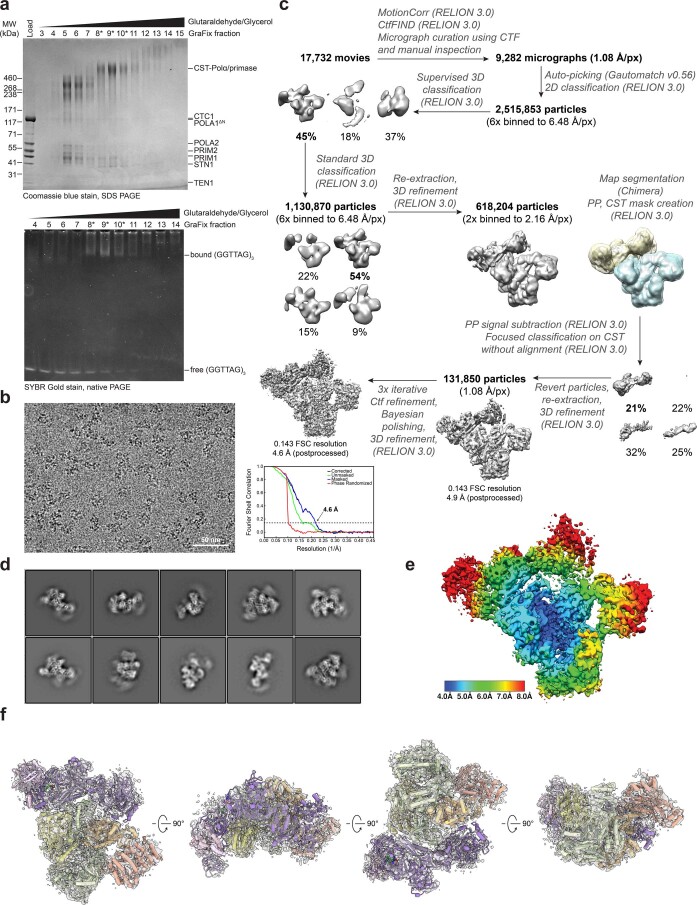
GraFix preparation of CST–PP^ΔN^ complex and analysis by cryo-EM. **a**, GraFix preparation of CST–PP^ΔN^–ssDNA. *Top:* Coomassie blue-stained SDS-PAGE gel (4–12% Tris-Glycine; Invitrogen) of GraFix fractions, showing formation of cross-linked CST–PP^ΔN^ species. Fractions marked with an asterisk (*) were pooled for analysis by cryo-EM. *Bottom:* SYBR Gold-stained native PAGE gel (4–20% TBE; Invitrogen) of GraFix fractions, showing free and bound ssDNA. This reconstitution was independently reproduced at least three times with similar results. **b**, Selected high defocus motion-corrected cryo-EM micrograph of CST–PP^ΔN^ (representative of 9,282 curated micrographs). **c**, Cryo-EM image-processing pipeline used for the CST–PP^ΔN^ complex, including supervised 3D classification and focused 3D classification steps used to select particles with intact CST. Gold-standard FSC curves for the final map of CST–PP^ΔN^ indicate a global resolution of 4.6 Å (FSC = 0.143). See Table 1 for full data collection and processing details. **d**, 2D-class averages obtained with the particle stack (131,850 particles) used to generate the final CST–PP^ΔN^ map show blurring at the peripheral regions, indicating flexibility. **e**, Local resolution estimates of the CST–PP^ΔN^ map. **f**, Orthogonal views of the CST–PP^ΔN^–ssDNA model fit into the cryo-EM map. Source data

The POLA1 subunit of Polα/primase contains a disordered N-terminal region (POLA1^N^, 1–335 aa) that is dispensable for catalysis and omitted in most structures of the enzyme^17,18^ (Fig. 1a). We purified Polα/primase lacking POLA1^N^ (referred to hereafter as PP^ΔN^), reconstituted a CST–PP^ΔN^(–ssDNA) complex (Extended Data Fig. 3a) as for full-length Polα/primase (referred to hereafter as PP^FL^), and collected cryo-EM data (Extended Data Fig. 3b). The omission of POLA1^N^ resulted in lower CST occupancy, so we introduced additional classification steps to select for particles containing intact CST (Extended Data Fig. 3c). We used 131,850 particles to generate the final map with a global resolution of 4.6 Å (Fig. 1b and Extended Data Fig. 3c,d). Local resolution estimates revealed lower resolution for the peripheral regions of CST and PP^ΔN^ (Extended Data Fig. 3e), likely due to flexibility, as suggested by the blurred-out regions in the 2D class averages (Extended Data Fig. 3d).

The crystal structure of apo PP^ΔN^ (PDB ID: 5EXR)^17^ and the cryo-EM structure of an ssDNA-bound CST monomer extracted from the decamer structure (PDB ID: 6W6W)^13^ could readily be docked into our density map. We then substituted the CTC1 structure with a model from the AlphaFold 2 database^19,20^, which provides information about the CTC1 N-terminus that was poorly resolved in the published CST cryo-EM map^13^. The ssDNA was included in the complex, as evidenced by the native gel of the GraFix fractions (Extended Data Fig. 3a), but no reliable density could be found for it owing to the low resolution of CST in the cryo-EM map. However, we observe low occupancy of the STN1 C-terminal half (STN1^C^, 184–398 aa) in that region (Fig. 1c) and, because ssDNA competes with STN1^C^ for that binding site^13^, we retained the 4 nt of ssDNA from the previously determined CST structure^13^ in the model we docked into our map (Fig. 1c).

After initial rigid-body docking followed by flexible fitting and refinement, the overall conformations of the two subcomplexes did not show major changes from their structures in isolation (Fig. 1c and Extended Data Fig. 3f). In the complex, Polα/primase remains in the occluded state^17^, in which the POLA2 subunit is blocking entry of DNA into the active site of POLA1 (Fig. 1c). This finding is consistent with reported results showing that cross-linking preferentially stabilizes the more compact, occluded state compared with the flexible, extended state of the enzyme^21,22^. Thus, we conclude that our structure likely captures a recruitment state of the complex that forms prior to active RNA and DNA synthesis by Polα/primase.

### Structural and evolutionary analysis of the CTC1–POLA1 interface

**Fig. 2:**
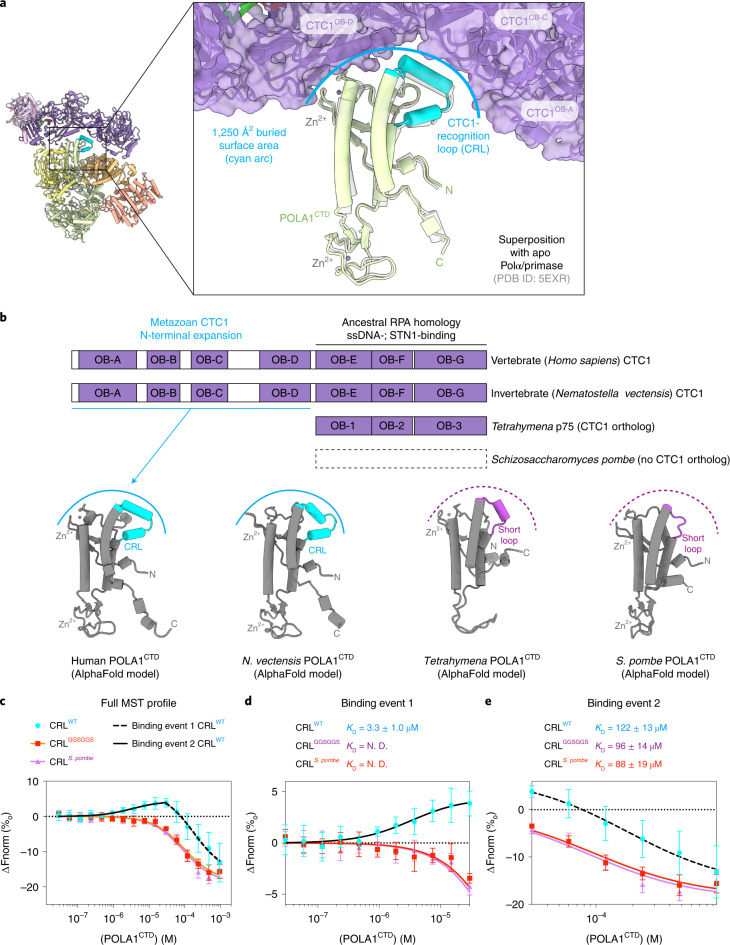
Structural analysis of the CTC1–POLA1^CTD^ interface. **a**, Zoomed-in view of the interaction of POLA1^CTD^ (cartoon) with CTC1 (cartoon and surface) superposed with POLA1^CTD^ from the apo Polα/primase structure (PDB ID: 5EXR)^17^. The CTC1-recognition loop (CRL, 1400–1424 aa) is highlighted in cyan in the cartoon representation. The corresponding cleft in CTC1 is indicated with a cyan arc. **b**, Comparison of POLA1^CTD^ models generated with AlphaFold 2 (refs. ^19,20^) from several species alongside domain comparisons of corresponding CTC1 orthologs. The metazoan CTC1 N-terminal expansion is indicated, and the binding pocket is represented by a cyan arc as in **a** or a dashed fuchsia arc in the non-metazoan species. **c**, Microscale thermophoresis measuring binding of POLA1^CTD^ proteins to RED-tris-NTA-labeled His_6_-MBP-CTC1–STN1–TEN1. Error bars represent s.e.m. for each data point, calculated from three independent thermophoresis measurements (Extended Data Fig. 6e). **d**,**e**, Zoom-in of binding events 1 and 2 from **c**, respectively. *K*_D_ values were calculated (N.D., not determined) with the MO Affinity Analysis (Nanotemper) software (split into two events for the CRL^WT^ and generated with all data for the CRL^GGSGGS^ and CRL^*S. pombe*^ mutants). *F*_norm_, normalized fluorescence. Source data

**Extended Data Fig. 4:**
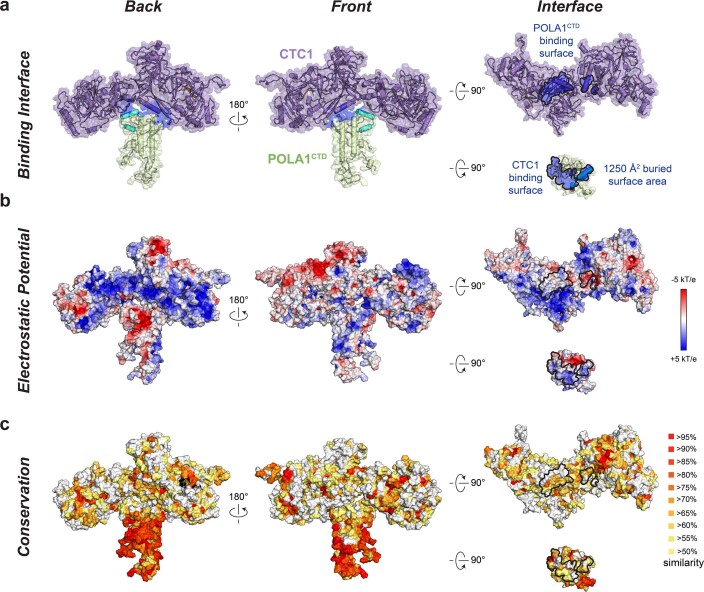
Analysis of the CTC1-POLA1^CTD^ interface surfaces. Surface representations of the CTC1-POLA1^CTD^ interface shown in two orientations related by a 180° rotation and views CTC1 and POLA1^CTD^ after separation to reveal the interface surfaces (outlined in black). **a**, Surface overlaid with cartoon representation of CTC1–POLA1^CTD^. Identification of CTC1 and POLA1^CTD^ binding surfaces, colored in dark blue. **b**, Surface representation colored according to electrostatic potential. **c**, Surface representation colored according to sequence conservation for metazoans using an alignment containing all species listed in Supplementary Table 1.

The primary interaction interface observed in our structure occurs between the C-terminal domain of POLA1 (POLA1^CTD^, 1,265–1,462 aa) and the N-terminal OB folds of CTC1 (Figs. 1c and 2a). The resolution is limiting for rigorous analysis of amino acid interactions, and analysis of surface electrostatic potential suggests that this interface is not driven by a dominant hydrophobic or charged interaction, but rather by shape complementarity of the two proteins, burying 1,250 Å^2^ of solvent-accessible surface area (Fig. 2a and Extended Data Fig. 4a,b).

**Extended Data Fig. 5:**
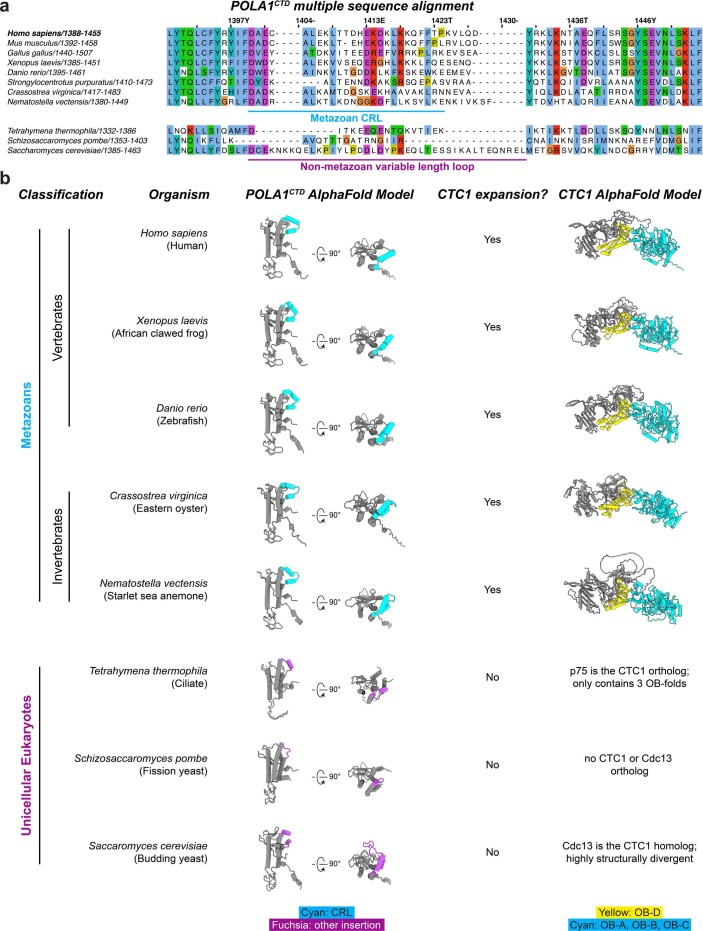
Conservation analysis of the CTC1-POLA1^CTD^ interface. **a**, Multiple sequence alignment showing the CRL region in POLA1. Residues are colored according to the color scheme used by Clustal X^43^. Accession codes for sequences are listed in Supplementary Table 1. **b**, Sequences for POLA1^CTD^ and CTC1 (when present) were modeled in AlphaFold 2^19,20^ and aligned in PyMOL (Schrödinger) (Supplementary Table 1). The CRL in metazoans is colored cyan, and the corresponding insertion region in unicellular eukaryotes is colored in fuchsia. For CTC1, the elongated OB-D is colored yellow and the rest of the N-terminus (OB-A, OB-B, OB-C, three-helix bundle) is colored cyan.

We identified a CTC1-recognition loop (CRL, 1400–1424 aa) in the POLA1^CTD^ that is shifted relative to its position in the apo structure^17^ to contact CTC1 (Fig. 2a). Sequence conservation analysis of the interface revealed that both CTC1 and the interacting region on the POLA1^CTD^ have low conservation at the primary sequence level (Extended Data Figs. 4c and 5a). However, the CRL is identifiable by an insertion of uniform length in metazoans, and we find that, when modeled using predictions from AlphaFold 2 (refs. ^19,20^), the CRL is structurally conserved in metazoans. Furthermore, we find that the presence of a CRL feature correlates with an expansion of metazoan CTC1 to contain the N-terminal OB folds that interact with the CRL. In unicellular eukaryotes, this loop diverges greatly between species and can be either shorter (for example, in *Tetrahymena thermophila* and *Schizosaccharomyces pombe*) or longer (for example, in *Saccharomyces cerevisiae*) and adopts a different predicted structure compared with that in the metazoan CRL (Fig. 2b and Extended Data Fig. 5b).

**Extended Data Fig. 6:**
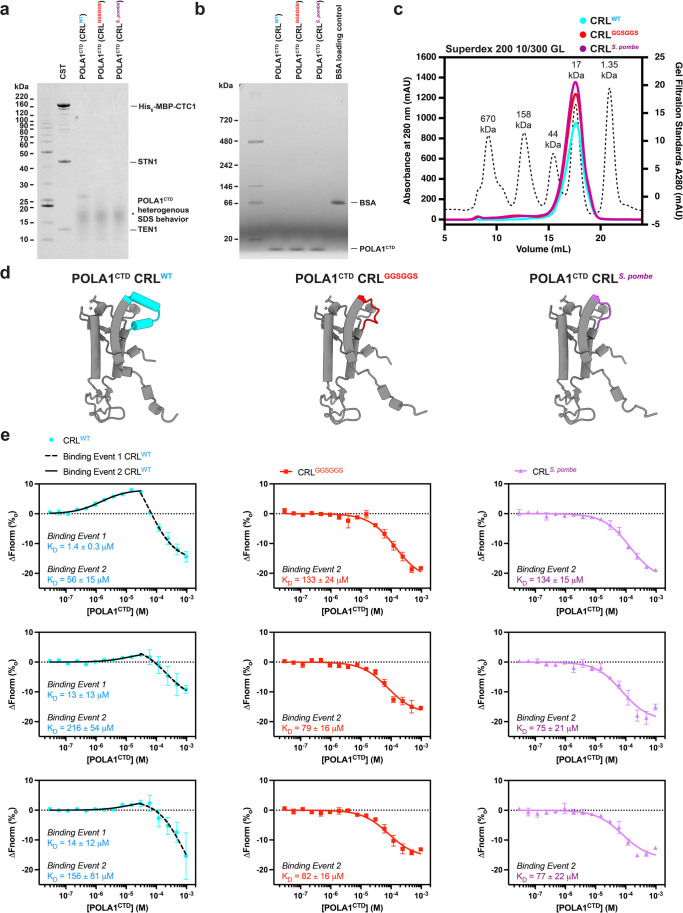
Purification and microscale thermophoresis analysis of POLA1^CTD^ CRL mutants. **a**, Coomassie blue-stained SDS-PAGE gels (4–12% (w/v) Bis-Tris, run in MES-SDS; Invitrogen) showing proteins used for MST analyses. POLA1^CTD^ constructs run as heterogeneously sized smears on SDS-PAGE. Gels are representative of three independent protein preparations. **b**, Native PAGE (4–16% Bis-Tris; Invitrogen) gel showing POLA1^CTD^ constructs and a 1 μg BSA loading control next to a NativeMark unstained protein standard. Gels are representative of three independent protein preparations. **c**, SEC-elution profiles of the POLA1^CTD^ constructs (estimated MW ~20 kDa) superposed with gel-filtration standards (BioRad). **d**, Models generated with AlphaFold 2^19,20^ of the human POLA1^CTD^ constructs encoding CRL^WT^ (cyan), CRL^GGSGGS^ (red), or CRL^*S. pombe*^ (fuchsia), demonstrating the accommodation of shortened linkers. **e**, Independent microscale thermophoresis experiments with POLA1^CTD^ constructs and RED-tris-NTA-labeled His_6_-MBP-CTC1–STN1–TEN1. Dissociation constant (K_D_) values were calculated with the MO Affinity Analysis (Nanotemper) software. Error bars represent the s.e.m. for each data point calculated from three capillary scans. The combined data are shown in Fig. 2c. Source data

To assess the involvement of the CRL in CST binding, we generated human POLA1^CTD^ constructs with the wild-type CRL (CRL^WT^, 1265–1462 aa), with the CRL swapped for a GGSGGS-linker (CRL^GGSGGS^, 1265–1402–GGSGGS–1423–1462 aa), or with the CRL swapped for the *S. pombe* short loop (CRL^*S. pombe*^, 1265–1399–QTTTGAT–1425–1462 aa) (Extended Data Fig. 6a–d). Although the POLA1^CTD^ constructs ran as heterogeneously sized smears in SDS–PAGE, they compressed to single bands in native PAGE (Extended Data Fig. 6a,b). Sharp symmetric peaks in SEC elution profiles (Extended Data Fig. 6c) and spectrophotometric (Nanodrop) quantification indicated that the protein was pure and free from nucleic acid contamination, respectively. We measured the affinity of the interaction between the POLA1^CTD^ constructs and fluorescently labeled CST using microscale thermophoresis (MST) (Fig. 2c). Two distinct binding events between the CRL^WT^ protein and CST were observed: the first binding event (1; Fig. 2d) is higher affinity and characterized by a positive change in the normalized fluorescence (Fig. 2d), and the second binding event (2) is lower affinity and characterized by a negative change in the normalized fluorescence (Fig. 2e). We separated the data on the basis of the two inflection points^23^ and calculated dissociation constant (*K*_D_) values of ~3.3 μM and ~122 μM for binding events 1 and 2, respectively. In contrast, the CRL^GGSGGS^ and CRL^*S. pombe*^ mutant proteins displayed binding in only the second event (Fig. 2c,d). For binding event 2, the determined *K*_D_ values for the CRL^GGSGGS^ and CRL^*S. pombe*^ proteins were ~96 μM and ~88 μM, respectively.

The CRL and one Zn^2+^-binding domain in POLA1^CTD^ form the module that fits into a complementary cleft in CTC1. We speculate that the high-affinity binding mode (event 1; Fig. 2d) observed in the MST experiments involves both the CRL and Zn^2+^-binding domain, whereas the lower affinity binding mode (event 2; Fig. 2e) reflects the interaction of the Zn^2+^-binding domain with CTC1, which is CRL-independent. In this second binding mode, CST could rock about the Zn^2+^-binding domain, echoing the hinge-like flexible motion of CST about POLA1 suggested by the cryo-EM data (Extended Data Fig. 3d,e). CTC1^OB-D^, an elongated OB fold that shares no homology with known OB folds^13^, forms the major interaction with POLA1^CTD^ (Fig. 2a and Extended Data Fig. 4). This finding is particularly interesting given the proposal that CST and Polα co-evolved in eukaryotes^24^. Our structure would then capture a metazoan-specific development in this trajectory.

Our structural model also places regions of CTC1 near POLA2 and PRIM2 (Fig. 1c). These two potential interaction sites are not well resolved in our cryo-EM map, but it is possible that these interactions are weak and/or more transient as CST flexes about the hinge generated by the POLA1^CTD^-CTC1 interaction.

### CST–Polα/primase maintains a 1:1 stoichiometry

**Fig. 3:**
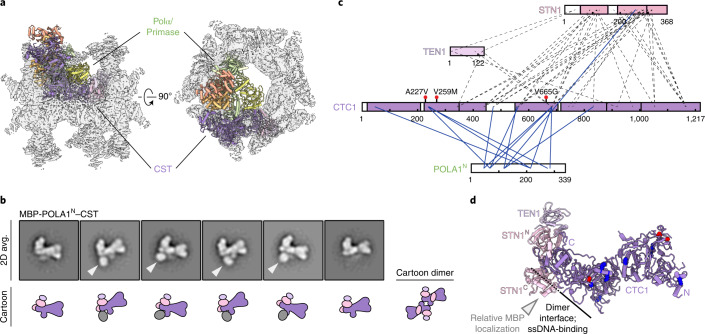
Mapping the CST-POLA1^N^ interaction and complex stoichiometry. **a**, CST–PP^ΔN^ structure (cartoon representation; colors as in Fig. 1) docked into the cryo-EM map^13^ of the CST decamer (transparent surface). **b**, Averages and cartoon representations of the six most populated RELION-3 2D classes of the MBP-POLA1^N^–CST complex. The MBP mass label is indicated with a white arrowhead when present. **c**, Cross-links between POLA1^N^ and CST subunits identified by CX-MS. Blue lines, cross-links between CST and POLA1^N^. Dashed lines, inter-subunit cross-links in CST. **d**, Cartoon representation of the CST monomer in the same view as in **b**. Lysine residues cross-linked to POLA1^N^ (**c**) are shown as blue spheres, and CP mutations previously characterized to disrupt Polα/primase binding^14^ are shown as red spheres. The white arrowhead indicates relative localization of the MBP mass label. Source data

**Extended Data Fig. 7:**
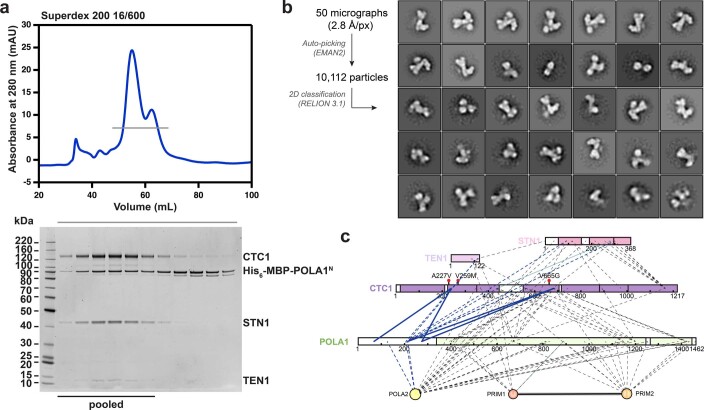
Reconstitution and analysis of the CST-POLA1^N^ interaction. **a**, SEC-elution profile of reconstituted His_6_-MBP-POLA1^N^–CST (top). The grey line indicates the fractions visualized on the SYPRO Ruby-stained SDS-PAGE gel (4–12% Bis-Tris, run in MOPS-SDS; Invitrogen) (bottom). The black bar indicates the fractions pooled for negative-stain EM and CX-MS analysis (see Fig. 3). This reconstitution was independently reproduced at least three times with similar results. **b**, Negative-stain EM image-processing pipeline and averages of the 35 (of 50) most populated classes of the reconstituted complex characterized in **a**. The size of the 128-px box corresponds to 358 Å^2^. **c**, Cross-links between POLA1^N^ and CST subunits identified by CX-MS analysis of the CST–PP^FL^ complex (Extended Data Fig. 1a). Blue solid lines: cross-links between CST and POLA1^N^ shared with the His_6_-MBP-POLA1^N^–CST dataset (Fig. 3c). Blue dashed lines: cross-links between CST and POLA1^N^ not shared with the His_6_-MBP-POLA1^N^–CST dataset (Fig. 2c). Black dashed lines: all other inter-subunit cross-links. Source data

The CST–Polα/primase complex is sterically incompatible with the previously reported ssDNA-bound CST decamer^13^, as it would bind in the center of the ring and sterically clash with neighboring CST subunits (Fig. 3a). CST has also been shown to dimerize upon ssDNA binding^13^, but two additional lines of evidence suggest that active CST–Polα/primase has a 1:1 stoichiometry. First, we do not observe CST dimers in our 2D class averages (Extended Data Figs. 1 and 2). Second, we characterized the CST-POLA1^N^ interaction to understand why CST–PP^FL^ did not yield a high-resolution map although PP^FL^ forms a more stable interaction with CST. We reconstituted a native complex of CST and MBP-tagged POLA1^N^ and analyzed it by negative-stain EM and cross-linking mass spectrometry (CX-MS) (Extended Data Fig. 7). With MBP as a mass label, we localized the N-terminus of POLA1^N^ to the primary CST dimerization interface (Fig. 3b). CX-MS analysis suggests that POLA1^N^ binds in multiple modes to CST, which could partially explain the heterogeneity observed with CST–PP^FL^ (Fig. 3c,d). It is possible that POLA1^N^ binding is restrained in the presence of the full complex, but we observe similar cross-links between CTC1 and POLA1^N^ in CX-MS analysis of CST–PP^FL^ (Extended Data Fig. 7c). Thus, we conclude that POLA1^N^ binds heterogeneously to CST in the region of the dimer interface, and the CST–Polα/primase fill-in machinery functions as a 1:1 complex.

### Coats plus mutations map to recruitment interfaces

**Fig. 4:**
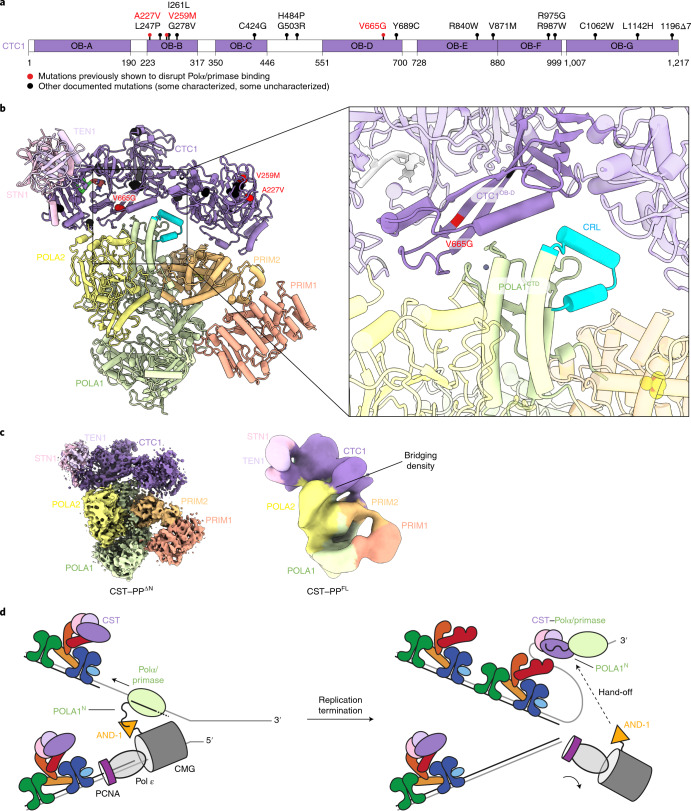
Disease mutation mapping and fill-in model. **a**, Summary of CP point mutations in CTC1. **b**, Mapping of mutations affecting Polα/primase binding on the CST–PP^ΔN^ structure. Colors as in **a**. Box, zoomed-in view of the primary CTC1^OB-D^-POLA1^CTD^ interaction. **c**, Comparison of the CST–PP^ΔN^ and CST–PP^FL^ (Extended Data Fig. 2a) cryo-EM density maps, segmented and colored according to docked CST and PP^ΔN^ models. **d**, Proposed model for the handoff of Polα/primase to shelterin-bound CST following telomere replication.

Three CP point mutations (p.A227V, p.V259M, and p.V665G) in CTC1 were previously described to disrupt Polα/primase association with CST^14,25^ (Fig. 4a). We mapped these residues onto our structure to investigate the molecular basis of dysfunction caused by these mutations (Fig. 4b). V665 resides on a β-strand of CTC1^OB-D^, so it is plausible that a glycine substitution would destabilize the β-sheet and OB fold, disrupting the primary interaction. The mutations at A227 and V259 reside on CTC1^OB-B^, which does not contact Polα/primase in the CST–PP^ΔN^ structure (Fig. 4b). However, one major difference we observe between the cryo-EM maps of CST–PP^ΔN^ and CST–PP^FL^ is the presence of connecting density between POLA2 and the CTC1 N-terminus, which we only observe when POLA1^N^ is present (Fig. 4c). Because cooperative binding between POLA1^N^ and POLA2^NTD^ (1–78 aa, attached by a flexible linker and not visualized in our CST–PP^ΔN^ structure) in other settings has been described^26^, we speculate that this bridging density is a combination of these two termini. Although our resolution is limited, this connection could potentially explain the CP mutations occurring at the CTC1 N-terminus (Fig. 4a–c).

## Discussion

In this study, we report a novel structure of the human CST–Polα/primase fill-in machinery. By chemically cross-linking the complex, we captured a conformational state that reveals how CST can recognize and bind the occluded state of Polα/primase through a newly uncovered interaction between CTC1 and POLA1^CTD^. We propose that this interaction is specific to metazoans and occurs during recruitment of Polα/primase to the telomere, prior to the start of RNA/DNA synthesis by the enzyme. Notably, this interface is formed by the N-terminal four OB folds of CTC1, a metazoan expansion of the subunit that further differentiates it from the paralogous RPA large subunit and from CTC1 homologs found in unicellular eukaryotes^13,24^. Our evolutionary conservation analysis identified a complementary species-specific loop in the POLA1^CTD^, termed the CRL, that appears to have co-evolved with the expansion in CTC1. Substitution of the CRL with a short loop, either a GGSGGS linker or the orthologous *S. pombe* sequence, abrogates binding in the high-affinity (*K*_D_ = ~1–10 μM) binding mode between POLA1^CTD^ and CST. MST experiments also captured an order-of-magnitude-lower affinity (*K*_D_ = ~100 μM) binding mode that is CRL-independent. The two binding modes are consistent with our cryo-EM structure, in which POLA1^CTD^ utilizes both the CRL and a Zn^2+^-binding motif to interact with CTC1.

Further work is needed to delineate the functional role of POLA1^N^ in regulating the fill-in machinery. We uncovered an interaction between POLA1^N^ and CST by using a combination of negative-stain EM, CX-MS, and cryo-EM with CST bound to POLA1^N^ in isolation and additionally bound in the context of PP^FL^. The CST–PP^FL^ complex associated more robustly than CST–PP^ΔN^, as inferred from higher CST occupancy in cryo-EM 2D averages and stability during native SEC. However, we observed greater structural heterogeneity in the cryo-EM data for CST–PP^FL^. The addition of POLA1^N^ may allow the complex to sample a greater conformational space, interfering with accurate alignment of the particles in the CST–PP^FL^ complex. POLA1^N^ is responsible for the flexible tethering of Polα/primase to the replisome via an interaction with AND-1 (refs. ^27,28^), and its extensive yet flexible interaction with CST suggests a potential spatiotemporal regulation of fill-in after telomere replication, where the replisome hands Polα/primase off to a shelterin-bound CST for C-strand synthesis (Fig. 4d).

Finally, our model informs on CP mutations (p.A227V, p.V259M, p.V665G) previously characterized to disrupt CST–Polα/primase association^14,25^. It is unlikely that these single point mutations result in complete loss of function, as such mutations would likely be lethal. Consistent with this framework of mild dysfunction, we observe that the three CP mutations map close to interaction interfaces but are not necessarily responsible for direct interaction with Polα/primase. For example, V665 resides on a β-strand in Ctc1^OB-D^ that is not directly on the interface, but the glycine substitution could destabilize the OB fold and weaken the interaction. Similarly, the Ctc1^OB-B^ mutations reside near the bridge observed in the CST–PP^FL^ cryo-EM map. Since those mutants (p.A227V, p.V259M, p.V665G) were characterized^14^, three more CP mutations have been reported in CTC1^OB-B^ (refs. ^29,30^). Presumably, these would be deleterious in a manner resembling that of p.A227V and p.V259M. Future genetic and functional studies are required to elucidate the precise mechanism by which these mutations cause CP, but our structural results provide a framework for understanding the molecular basis of human disease linked to CST–Polα/primase, its role in telomere maintenance, and its contribution to DSB repair.

## Methods

### DNA construct and baculovirus generation

DNA fragments encoding human CTC1, STN1, TEN1, POLA1 (POLA1^N^, 1–339 aa; POLA1^FL^, 1–1462 aa; POLA1^ΔN^, 335–1492 aa), POLA2, PRIM1, and PRIM2 were cloned using Gibson assembly into the biGBac vector system^31^ with affinity tags. The plasmids used in this study are pBIG1a(His_6_-MBP-PreSc-CTC1/STN1/TEN1), pBIG2ab((His_6_-MBP-PreSc-POLA1^FL^/POLA2)/(PRIM1/StrepII-TEV-PRIM2)), pBIG2ab((His_6_-MBP-PreSc-POLA1^ΔN^/POLA2)/(PRIM1/StrepII-TEV-PRIM2)), and pLIB(His_6_-MBP-PreSc-POLA1^N^). Recombinant bacmids were generated from these plasmids using MAX Efficiency DH10Bac competent cells (Gibco; cat. no. 10361012) and transfected into Sf9 insect cells (Gibco; cat. no. 11496015) with Cellfectin II Reagent (Gibco) to generate a P1 baculovirus stock. P1 baculovirus was amplified in adherent Sf9 insect cells to generate P2 and P3 stocks, and the P3 virus was used to infect Tni suspension insect cell culture (Expression Systems; cat. no. 94-002S) for protein expression.

Constructs for expression of POLA1^CTD^ in *E. coli* (CRL^WT^: 1265–1462 aa; CRL^GGSGGS^: 1265–1402–GGSGGS–1423–1462 aa; CRL^*S. pombe*^: 1265–1399– QTTTGAT–1425–1462 aa) were cloned with an N-terminal His_6_-Smt3 tag into a pRSFDuet-1 vector in the first multiple cloning site. Mutants were generated by Gibson assembly and confirmed by Sanger sequencing.

### Protein expression and purification

Fifty milliliters of P3 baculovirus were used per 500 mL of Tni culture, infected at a cell density of ~2 ×10^6^ cells/mL. The infected cells were grown in spinner flasks at 150 r.p.m. for 72 h at 27 °C. Cells were collected by centrifugation at 1,000*g* and transferred to a syringe before droplets were flash frozen in liquid nitrogen. The frozen pellets were lysed by cryogenic milling (Retsch) and the cryo-milled powder was resuspended in a buffer containing 20 mM Tris (pH 8.0), 500 mM NaCl, 15 mM 2-mercaptoethanol (β-ME), 20 mM imidazole, 10% (v/v) glycerol, and 1 mM phenylmethylsulfonyl fluoride (PMSF), supplemented with cOmplete EDTA-free protease inhibitor cocktail (Roche). The lysate was cleared by centrifugation at 4 °C and 40,000*g* for 1 h. Supernatants were incubated with end-over-end rotation for 1 h at 4 °C with Ni-NTA resin (Invitrogen) equilibrated with a buffer containing 20 mM Tris (pH 8.0), 500 mM NaCl, 15 mM β-ME, 20 mM imidazole, and 5% glycerol and subsequently washed with 20–50 column volumes (CV) of the same buffer. Bound protein was eluted in a buffer containing 20 mM HEPES (pH 7.5), 150 mM NaCl, 0.5 mM tris(2-carboxyethyl)phosphine (TCEP), 250 mM imidazole, and 5% glycerol.

After elution from the Ni-NTA resin, His_6_-MBP-PreSc-CTC1/STN1/TEN1 was incubated with rhinovirus 3C protease overnight at 4 °C to remove the His_6_-MBP tag. The cleaved protein was loaded onto a HiTrap Heparin HP column (GE Healthcare) equilibrated with a buffer containing 20 mM HEPES (pH 7.5), 150 mM NaCl, 0.5 mM TCEP, and 5% glycerol and eluted with a linear gradient of NaCl concentration to 1 M. Protein-containing fractions were loaded onto a HiLoad Superdex 200 16/600 PG column (GE Healthcare) equilibrated with a buffer containing 20 mM HEPES (pH 7.5), 300 mM NaCl, 0.5 mM TCEP, and 5% glycerol. For microscale thermophoresis experiments, the protease-cleavage step was omitted and the His_6_-MBP-PreSc-CTC1/STN1/TEN1 protein eluted from the Ni-NTA column was directly subjected to Heparin-affinity and size-exclusion chromatography as described above.

After elution from the Ni-NTA resin, His_6_-MBP-PreSc-POLA1^FL^/POLA2/PRIM1/StrepII-TEV-PRIM2 and His_6_-MBP-PreSc-POLA1^ΔN^/POLA2/PRIM1/StrepII-TEV-PRIM2 were loaded onto a RESOURCE Q column (Cytiva) equilibrated with a buffer containing 20 mM HEPES (pH 7.5), 150 mM NaCl, 0.5 mM TCEP, and 5% glycerol, and were eluted with a linear gradient of NaCl concentration to 0.5 M. Protein-containing fractions were loaded onto a HiLoad Superdex 200 16/600 PG column equilibrated with a buffer containing 20 mM HEPES (pH 7.5), 150 mM NaCl, 0.5 mM TCEP, and 5% glycerol.

After elution from the Ni-NTA resin, His_6_-MBP-PreSc-POLA1^N^ was directly loaded onto a HiLoad Superdex 200 16/600 PG column equilibrated with a buffer containing 20 mM HEPES (pH 7.5), 150 mM NaCl, 0.5 mM TCEP, and 5% glycerol.

His_6_-Smt3 tagged POLA1^CTD^ proteins were expressed in *E. coli* Rosetta(DE3) cells (Novagen; cat. no. 70954-3) in Super Broth medium (Teknova) and induced at an OD_600_ of ~0.8 with 0.5 mM IPTG (Soltec). Cells were collected by centrifugation at 4,000*g* and were resuspended in a buffer containing 20 mM Tris (pH 8.0), 500 mM NaCl, 4 mM β-ME, 20 mM imidazole, and 10% glycerol, supplemented with complete EDTA-free protease inhibitor cocktail (Roche) and flash frozen in liquid nitrogen. Cells were supplemented with 20 μg/mL DNase I (Sigma), 20 μg/mL RNase A (Sigma), 20 μg/mL lysozyme (Sigma), 1 mM CaCl_2_, and 1 mM MgCl_2_, lysed by sonication, and centrifuged at 4 °C and 40,000*g* for 1 h. Supernatants were filtered through a 0.45-μm syringe filter (Millipore), incubated with end-over-end rotation for 1 h at 4 °C with Ni-NTA resin (Invitrogen) equilibrated with a buffer containing 20 mM Tris (pH 8.0), 500 mM NaCl, 4 mM β-ME, and 20 mM imidazole, and were subsequently washed with 20–50 CV of the same buffer. Bound protein was eluted with 250 mM imidazole in a buffer containing 20 mM HEPES (pH 7.5), 150 mM NaCl, and 0.1 mM TCEP. The His_6_-Smt3 tag was removed by cleavage with His_6_-Ulp1 protease concurrent with overnight dialysis into buffer containing 20 mM HEPES (pH 7.5), 150 mM NaCl, and 0.1 mM TCEP. Dialyzed protein was incubated with end-over-end rotation for 1 h at 4 °C with Ni-NTA resin (Invitrogen) equilibrated with dialysis buffer to rebind the His_6_-Smt3, His_6_-Ulp1, and any uncleaved protein. The flowthrough (containing cleaved POLA1^CTD^ protein) was collected, concentrated, and loaded onto a Superdex 200 10/300 GL column equilibrated with a buffer containing 20 mM HEPES (pH 7.5), 150 mM NaCl, and 0.1 mM TCEP.

Unless otherwise stated, all proteins were concentrated, flash frozen in liquid nitrogen, and stored in aliquots at -80 °C.

### Reconstitution of native CST–PP^FL^ and MBP-POLA1^N^–CST

Freshly purified PP^FL^ and CST were mixed in equimolar amounts and incubated for 1 h at 4 °C prior to loading onto a HiLoad Superdex 200 16/600 PG column equilibrated with a buffer containing 20 mM HEPES (pH 7.5), 150 mM NaCl, 0.5 mM TCEP, and 5% glycerol. The indicated fractions (Extended Data Fig. 1a) were pooled and concentrated. Purified CST was mixed with a twofold molar excess of His_6_-MBP-PreSc-POLA1^N^ and incubated for 1 h at 4 °C prior to being loaded onto a HiLoad Superdex 200 16/600 PG column equilibrated with a buffer containing 20 mM HEPES (pH 7.5), 150 mM NaCl, 0.5 mM TCEP, and 5% glycerol. The indicated fractions (Extended Data Fig. 5) were pooled and concentrated.

### Negative-stain EM sample preparation, data collection, and image processing

Protein samples for negative-stain EM (3.5-μL drops, in a concentration range of 0.01–0.05 mg/mL) were adsorbed to glow-discharged carbon-coated copper grids with a collodion film, washed with three drops of deionized water, and stained with two drops of freshly prepared 0.7% w/v uranyl formate. Samples were imaged at room temperature using a Phillips CM10 electron microscope equipped with a tungsten filament and operated at an acceleration voltage of 80 kV. The magnification used corresponds to a calibrated pixel size of 2.8 Å. Particle coordinates were auto-picked using the Swarm picker in EMAN2 (ref. ^32^). Particle extraction and 2D classification were performed in RELION 3.0 or RELION 3.1, as indicated.

### GraFix stabilization of CST–Polα/primase complexes

Purified CST and Polα/primase (PP^FL^ or PP^ΔN^) were first mixed in equimolar amounts (for a 1.5 μM final protein concentration of each component) and incubated on ice for 1 h before the addition of 5′-(GGTTAG)_3_-3′ ssDNA at a 2.25-fold molar excess. The protein–ssDNA mixture was incubated on ice for 2 h and then loaded on top of a 11-mL linear 10%–30% glycerol and 0–5 mM glutaraldehyde GraFix gradient in 20 mM HEPES, pH 7.5, 150 mM NaCl, 10 mM MgCl_2_, and 0.5 mM TCEP. Centrifugation was carried out at 288,000*g* (41,000 r.p.m.) in an SW41 Ti rotor (Beckman Coulter) for 18 h at 4 °C. The top 3 mL of the gradient was discarded and then 500-μL fractions were manually collected from the top of the gradient and quenched with Tris-HCl (pH 7.5) at a final concentration of 50 mM. Fractions containing CST–Polα/primase were concentrated and diluted three times in an Amicon Ultra concentrator (Millipore; 30-kDa cut-off) with glycerol- and glutaraldehyde-free buffer (20 mM HEPES pH 7.5, 150 mM NaCl, 10 mM MgCl_2_, 0.5 mM TCEP). The concentrated samples were checked first with negative-stain EM for homogeneity prior to cryo-EM specimen preparation.

### Cryo-EM sample preparation and data collection

Four microliters of the GraFix-stabilized samples were applied to Quantifoil R 1.2/1.3 mesh Au400 holey carbon grids covered with a graphene oxide support layer (EMS), blotted for 1–1.5 sec, and plunge frozen in liquid ethane using a Vitrobot Mark IV (Thermo Fisher Scientific) operated at 4 °C and 100% humidity.

Cryo-EM imaging was performed in the Cryo-EM Resource Center at the Rockefeller University using SerialEM^33^. Data-collection parameters are summarized in Table 1.

For CST–PP^FL^, one dataset (dataset 1) was collected on a 200-kV Talos Arctica electron microscope (Thermo Fisher Scientific) at a nominal magnification of ×28,000 (TEM nanoprobe), corresponding to a calibrated pixel size of 1.5 Å on the specimen level. Images were collected using a defocus range from −1.5 to −3 μm with a K2 Summit direct electron detector (Gatan) in super-resolution counting mode. Exposures of 10 sec were dose-fractionated into 50 frames (200 ms per frame) with a dose rate of 12 electrons/pixel/sec (approximately 1.07 electrons per Å^2^ per frame), resulting in a total dose of 53 electrons per Å^2^. The second dataset (dataset 2) was collected on a 300-kV Titan Krios electron microscope at a nominal magnification of ×53,000 (EFTEM nanoprobe), corresponding to a calibrated pixel size of 1.32 Å on the specimen level. Images were collected using a defocus range from -1 to -2.2 μm with a K3 direct electron detector (Gatan) in super-resolution counting mode. Exposures of 3 sec were dose-fractionated into 50 frames (60 ms per frame) with a dose rate of 33 electrons/pixel/sec (approximately 1.14 electrons per Å^2^ per frame), resulting in a total dose of 57 electrons per Å^2^.

For CST–PP^ΔN^, data were collected on a 300-kV Titan Krios electron microscope at a nominal magnification of ×64,000, corresponding to a calibrated pixel size of 1.08 Å on the specimen level. Images were collected using a defocus range from –1 to –2.2 μm with a K3 direct electron detector (Gatan) in super-resolution counting mode. Exposures of 3 sec were dose-fractionated into 50 frames (60 ms per frame), with a dose rate of 30 electrons/pixel/sec (approximately 1.03 electrons per Å^2^ per frame), resulting in a total dose of 52 electrons per Å^2^.

### Cryo-EM data processing

For all datasets, movie stacks were motion-corrected with the RELION-3 (ref. ^34^) implementation of MotionCor2 and motion-corrected micrographs were manually inspected and curated (graphene oxide coverage of grids were inconsistent) prior to CTF parameter estimation with CTFFIND4 (ref. ^35^) implemented in RELION-3. Particles were automatically picked with Gautomatch and extracted in RELION-3 for all further 2D and 3D processing steps. Auto-picked particles were examined by 2D classification, and particles in ‘bad’ classes corresponding to ice contamination or graphene oxide fold lines were discarded. The first reference model was generated using RELION-3’s 3D initial model job and subsequently improved as continued 3D classification produced better maps.

For the CST–PP^FL^ complex, multiple processing strategies were pursued to generate higher-resolution maps but were unsuccessful owing to substantial heterogeneity among the particles. The reported standard image-processing pipeline was performed with twofold binned images (to speed up computation), as the resolution of the resulting map did not approach the Nyquist limit. Because the two datasets were collected on different microscopes, RELION 3.1 was used to combine the particle stacks (imported as two separate optics groups). The combined particles were used for further 3D classification and 3D refinement (Extended Data Fig. 2).

For the CST–PP^ΔN^ complex, a supervised 3D classification step in RELION-3 was introduced, using three references: CST–PP^ΔN^, PP^ΔN^ alone, and a noise/junk ‘decoy’ class. After discarding particles assigned to the latter two classes, the remaining particles were subjected to 3D classification into four classes using a single reference. Because particles in the class with the best-defined features still showed low CST occupancy, a consensus 3D refinement was performed and the resulting map segmented into CST and PP^ΔN^ using UCSF Chimera^36^. Using a mask generated in RELION-3, the partial signal of PP^ΔN^ was subtracted and the signal-subtracted particles were subjected to focused 3D classification without alignment. The final stack contained 131,850 particles showing intact CST. The particles were reverted to include PP^ΔN^, and used for three cycles of iterative 3D refinement, CTF refinement, and Bayesian polishing. The resulting density map was sharpened by post-processing, and Fourier shell correlation (FSC) curves and a local resolution map were calculated in RELION-3 (Extended Data Fig. 3).

### Model building and refinement

An atomic model was built into the 4.6-Å resolution map of the CST–PP^ΔN^ complex. The crystal structure of the apo PP^ΔN^ (PDB ID: 5EXR) and cryo-EM structure of CST (PDB ID: 6W6W) were used for initial rigid-body docking into the map. The map showed only weak density corresponding to the C-terminal half of STN1 (STN1^C^, 184–368 aa), likely owing to the ssDNA charge preventing STN1^C^ binding, as previously described^13^, and was therefore removed for model building. The geometry of PRIM1 in the PP^ΔN^ crystal structure is poor and it was substituted in our initial model with a crystal structure of PRIM1 determined at higher resolution (PDB ID: 6RB4, alternate conformers removed^37^). Although the geometries of the other PP subunits in the starting model (PDB ID: 5EXR) were also poor, we chose to continue with this model because it is the only experimentally determined model of apo POLA1/POLA2/PRIM2 in the occluded conformation and retains inter-subunit contact information that would not be captured by AlphaFold 2 models or other experimental structures of the subunits in isolation. The N-terminus of CTC1 was poorly resolved in the previously determined cryo-EM map and not modeled, so it was substituted with the AlphaFold 2 model of CTC1 (AF-Q2NKJ3-F1), which agrees with the experimentally determined structure of CTC1 (refs. ^19,20^). All subunits were docked into the cryo-EM map using the Chimera ‘Fit in Map’ tool, and the model was flexibly fitted using the ISOLDE plugin for ChimeraX^38,39^. After the model was improved by iterative cycles of refinement in Phenix (phenix.real_space_refine) and manual adjustment in Coot^40^, the geometry and map fit of the final model was validated (phenix.validation_cryoem)^41^.

### Evolutionary conservation analysis

All sequence accession numbers used in this study are listed in Supplementary Table 1. Protein sequences obtained from BLAST (blastp suite) were analyzed using Jalview^42,43^ and multiple sequence comparison by log expectation (MUSCLE). Representative sequences for CTC1 and POLA1 were modeled in AlphaFold 2 (refs. ^19,20^) with the template database from 14 May 2020 and the casp14 preset. AlphaFold models were aligned in PyMOL (Schrödinger) and visualized in ChimeraX^39^.

### Microscale thermophoresis

Microscale thermophoresis experiments were performed on a NanoTemper Monolith NT.115 machine. All samples were buffer exchanged in centrifugal concentrators (Amicon Ultra-0.5 mL) into buffer containing 20 mM HEPES (pH 7.5), 150 mM NaCl, 5 mM DTT, and 0.05% (v/v) Tween-20. His_6_-MBP-CST was labeled according to manufacturer instructions with RED-tris-NTA second-generation dye (NanoTemper Technologies). Fifty nanomolar labeled His_6_-MBP-CST was incubated with serial dilutions of unlabeled POLA1^CTD^ constructs, and thermophoresis was measured at room temperature with an excitation power of 20% and an MST power of 20%. Titrations were performed in triplicate (experimental replicates), and capillary scans were performed in triplicate (technical replicates). Data were analyzed at the 10-sec time point with the MO Affinity Analysis Software (version 2.3, NanoTemper Technologies) using the *K*_D_ fit option with outliers owing to aggregation automatically determined by the software. For CRL^WT^, the data were manually split to account for the presence of two binding events^23^. Reported *K*_D_ values were calculated in the MO Affinity Analysis software and data were plotted with Prism 9 (GraphPad).

### In vitro cross-linking

BS3 cross-linker (Proteochem, c1103) was dissolved in LC–MS-grade H_2_O (Proteochem, LC6330) at 50 mM. DSS cross-linker (Proteochem, c1105) was dissolved in oxygen-depleted DMSO (Millipore-Sigma, 900645) to 100 mM stock concentration. Cross-linker was added to the target complex (BS3: CST–POLA1^N^; DSS: CST–PP^FL^) prepared in NHS-ester non-reactive buffer (1 mg/mL) to the final concentration of 0.35–0.75 mM. Reactions were performed at 25 °C in disposable inert cuvettes (UVette, Eppendorf), and monitored by continuous looped dynamic light scattering measurements of polydispersity^44^ (Pd < 10%; DynaPro NanoStar, Wyatt). Cross-linking was quenched after 30 min of incubation by addition of Tris-HCl (pH 8.0) to a final concentration of 5 mM.

### Mass spectrometry and data analysis

Samples were dialyzed against 100 mM ammonium bicarbonate, reduced with 50 mM TCEP at 60 °C for 10 min and alkylated with 50 mM iodoacetamide in the dark for 15 min at 37 °C. Digestion was carried out at 37 °C overnight with 125 ng/mL sequencing-grade modified trypsin (Fisher Scientific, PI90057) in 25 mM ammonium bicarbonate supplemented with ProteaseMax (Fisher Scientific, PRV2071). Reaction mix was supplemented with trifluoroacetic acid (TFA, Fisher Scientific, A116) to a final concentration of 0.1%. The resulting peptides were passed through C18 Spin Tips (Fisher Scientific, PI84850) before elution with 40 μL of 80% acetonitrile (ACN, Fisher Scientific, A9561) in 0.1% TFA. Eluted peptides were dried and resuspended in 20 μL 0.1% formic acid (FA; Fisher Scientific, A117) for MS analysis. Peptides were analyzed in an Orbitrap Fusion Lumos mass spectrometer (Thermo Scientific) coupled to an EASY-nLC (Thermo Scientific) liquid chromatography system, with a 2 μm, 500 mm EASY-Spray column. The peptides were eluted over a 120-min linear gradient from 96% buffer A (0.1 % FA in water) to 40% buffer B (0.1 % FA in ACN), then continued to 98% buffer B over 20 min with a flow rate of 250 nL/min. Each full MS scan (*R* = 60,000) was followed by 20 data-dependent MS2 scans (*R* = 15,000) with high-energy collisional dissociation (HCD) and an isolation window of 2.0 *m/z*. Normalized collision energy was set to 35. Precursors of charge state ≤ 3 were collected for MS2 scans in enumerative mode, precursors of charge state 4–6 were collected for MS2 scans in cross-link discovery mode (both were performed for each sample); monoisotopic precursor selection was enabled and a dynamic exclusion window of 30.0 sec was set. Raw files obtained in enumerative mode were analyzed by pFind3 software^45^ in open search mode and protein modifications inferred by pFind3 and comprising >0.5% of total protein were included as the variable modifications in pLink2 (ref. ^46^) search parameters. pLink2 results were filtered for FDR (<5%), *e*-value (<1 × 10^–3^), score (<1 × 10^–2^), and abundance (PSMs ≥ 5). Cross-links were visualized using xiNET^47^.

### Reporting Summary

Further information on research design is available in the Nature Research Reporting Summary linked to this article.

## Online content

Any methods, additional references, Nature Research reporting summaries, source data, extended data, supplementary information, acknowledgements, peer review information; details of author contributions and competing interests; and statements of data and code availability are available at 10.1038/s41594-022-00766-y.

## Supplementary information

Supplementary Information Supplementary Table 1

Reporting Summary

## Data Availability

Starting models used in this study can be found in the Protein Data Bank under the accession codes PDB 6W6W, PDB 5EXR, and PDB 6RB4 and in the AlphaFold Protein Structure Database under accession code AF-Q2NKJ3-F1. The cryo-EM maps generated in this study have been deposited at the Electron Microscopy Data Bank under accession codes EMD-26346 (CST–PP^ΔN^) and EMD-26347 (CST–PP^FL^), and the CST–PP^ΔN^ coordinates have been deposited in the Protein Data Bank under accession code PDB 7U5C. Source data are provided with this paper.

## Source data

Source Data Fig. 2 Statistical Source Data
Source Data Fig. 3 Statistical Source Data
Source Data Extended Data Fig. 1 Unprocessed Gels
Source Data Extended Data Fig. 3 Unprocessed Gels, Uncropped Micrograph
Source Data Extended Data Fig. 6 Unprocessed Gels
Source Data Extended Data Fig. 6 Statistical Source Data
Source Data Extended Data Fig. 7 Unprocessed Gels
Source Data Extended Data Fig. 7 Statistical Source Data
