# Supplementary material for: Cryo-EM structure of the human CST–Polα/primase complex in a recruitment state
Source: Nat Struct Mol Biol. 2022 May 16;29(8):813–9. doi: 10.1038/s41594-022-00766-y (PMC9371972; doi:10.1038/s41594-022-00766-y)
Supplement: Supplementary file 1 — Supplementary Table 1 [file 41594_2022_766_MOESM1_ESM.pdf]

nature portfolio

https://doi.org/10.1038/s41594-022-00766-y

Supplementary information

Cryo-EM structure of the human CST–Polα/primase complex in a recruitment state

In the format provided by the authors and unedited

Supplementary Table 1. Accession numbers of the genes used in evolutionary conservation analysis.

Gene	Organism	Accession Number
CTC1	*Homo sapiens*	NP_079375.3
CTC1	*Pan troglodytes*	XP_024206271.1
CTC1	*Mus musculus*	NP_001013274.2
CTC1	*Rattus norvegicus*	NP_001100480.1
CTC1	*Gallus gallus*	XP_015150604.3
CTC1	*Anolis carolinensus*	XP_003227344.3
CTC1	*Alligator mississippiensis*	XP_019331858.1
CTC1	*Xenopus laevis*	NP_001233242.1
CTC1	*Takifugu rubripes*	XP_029695834.1
CTC1	*Danio rerio*	NP_001071213.1
CTC1	*Callorhinchus milii*	XP_007906537.1
CTC1	*Branchiostoma floridae*	XP_035665808.1
CTC1	*Crassostrea virginica*	XP_022334778.1
CTC1	*Nematostella vectensis*	XP_032239384.1
POLA1	*Homo sapiens*	NP_058633.2
POLA1	*Pan troglodytes*	XP_016798596.1
POLA1	*Mus musculus*	NP_032918.1
POLA1	*Rattus norvegicus*	NP_445931.1
POLA1	*Gallus gallus*	XP_040513999.1
POLA1	*Anolis carolinensus*	XP_008105544.1
POLA1	*Alligator mississippiensis*	XP_014451207.1
POLA1	*Xenopus laevis*	NP_001082055.1
POLA1	*Takifugu rubripes*	XP_003967924.1
POLA1	*Danio rerio*	NP_001292393.1
POLA1	*Callorhinchus milli*	XP_042190336.1
POLA1	*Branchiostoma floridae*	XP_035693536.1
POLA1	*Crassostrea virginica*	XP_022344658.1
POLA1	*Nematostella vectensis*	XP_032236096.1
