# Supplementary material for: Cryo-EM structure of the human CST–Polα/primase complex in a recruitment state
Source: Nat Struct Mol Biol. 2022 May 16;29(8):813–9. doi: 10.1038/s41594-022-00766-y (PMC9371972; doi:10.1038/s41594-022-00766-y)
Supplement: Supplementary file 2 — Reporting Summary [file 41594_2022_766_MOESM2_ESM.pdf]

nature portfolio

Corresponding author(s): Titia de Lange, delange@rockefeller.edu

Last updated by author(s): March 29, 2022

Reporting Summary

Nature Portfolio wishes to improve the reproducibility of the work that we publish. This form provides structure for consistency and transparency in reporting. For further information on Nature Portfolio policies, see our Editorial Policies and the Editorial Policy Checklist.

Statistics

For all statistical analyses, confirm that the following items are present in the figure legend, table legend, main text, or Methods section.

n/a	Confirmed	
☐	☒	The exact sample size (*n*) for each experimental group/condition, given as a discrete number and unit of measurement
☐	☒	A statement on whether measurements were taken from distinct samples or whether the same sample was measured repeatedly
☒	☐	The statistical test(s) used AND whether they are one- or two-sided<br>*Only common tests should be described solely by name; describe more complex techniques in the Methods section.*
☐	☒	A description of all covariates tested
☒	☐	A description of any assumptions or corrections, such as tests of normality and adjustment for multiple comparisons
☐	☒	A full description of the statistical parameters including central tendency (e.g. means) or other basic estimates (e.g. regression coefficient) AND variation (e.g. standard deviation) or associated estimates of uncertainty (e.g. confidence intervals)
☒	☐	For null hypothesis testing, the test statistic (e.g. *F*, *t*, *r*) with confidence intervals, effect sizes, degrees of freedom and *P* value noted<br>*Give P values as exact values whenever suitable.*
☒	☐	For Bayesian analysis, information on the choice of priors and Markov chain Monte Carlo settings
☒	☐	For hierarchical and complex designs, identification of the appropriate level for tests and full reporting of outcomes
☒	☐	Estimates of effect sizes (e.g. Cohen's *d*, Pearson's *r*), indicating how they were calculated

Our web collection on statistics for biologists contains articles on many of the points above.

Software and code

Policy information about availability of computer code

Data collection	SerialEM v3.6, Nanotemper MO Affinity Control v2.3
Data analysis	Relion 3.0, Relion 3.1, Gautomatch 0.56, Ctffind 1.08, UCSF Chimera 1.13, Phenix 1.17.1, UCSF ChimeraX 0.93, EMAN 2.1, Nanotemper MO Affinity Analysis v2.3, pFind3, xiNET (http://crosslinkviewer.org/), PyMOL v2.1.1, GraphPad Prism v9.3.1, AlphaFold v2.0, Jalview 2.11.1.0, MUSCLE (https://www.ebi.ac.uk/Tools/msa/muscle/)

For manuscripts utilizing custom algorithms or software that are central to the research but not yet described in published literature, software must be made available to editors and reviewers. We strongly encourage code deposition in a community repository (e.g. GitHub). See the Nature Portfolio guidelines for submitting code & software for further information.

Data

Policy information about availability of data

All manuscripts must include a data availability statement. This statement should provide the following information, where applicable:

- Accession codes, unique identifiers, or web links for publicly available datasets
- A description of any restrictions on data availability
- For clinical datasets or third party data, please ensure that the statement adheres to our policy

Starting models used in this study can be found in the Protein Data Bank under the accession codes PDB-6W6W, PDB-5EXR, and PDB 6RB4 and in the AlphaFold Protein Structure Database under accession code AF-Q2NKJ3-F1. The cryo-EM maps generated in this study have been deposited at the Electron Microscopy Data Bank under accession codes EMD-26346 (CST•PP[illegible]N) and EMD-26347 (CST•PPFL), and the CST•PP[illegible]N coordinates have been deposited in the Protein Data Bank under accession code PDB-7U5C. Source data for Figures 2, 3, and Extended Data Figures 1, 3, 6, and 7 are provided with this paper.

March 2021

Field-specific reporting

Please select the one below that is the best fit for your research. If you are not sure, read the appropriate sections before making your selection.

☒ Life sciences ☐ Behavioural & social sciences ☐ Ecological, evolutionary & environmental sciences

For a reference copy of the document with all sections, see nature.com/documents/nr-reporting-summary-flat.pdf

Life sciences study design

All studies must disclose on these points even when the disclosure is negative.

Sample size	Sample sizes for the Cryo-EM datasets were determined by the need to obtain meaningful structures and the availability of cryo-EM time. For the CST-PP-FL dataset, 6340 movie stacks were collected and 109,224 particles were used for the final reconstruction. For the CST-PP-deltaN dataset, 17732 movie stacks were collected and 131,850 particles were used for the final reconstruction, which was sufficient to yield a 4.6-Å resolution structure. For other experiments, no sample-size determination was performed, as the biochemical experiments followed standard practices.
Data exclusions	Micrographs clearly suffering from astigmatism, image drift, poor graphene oxide coverage, ice contamination, and/or cubic ice formation were excluded during the micrograph curation step in all cryo-EM datasets analyzed. Particles in 2D classes showing no secondary structural features and in 3D classes showing unsatisfactory structural features were excluded from the final reconstructions in all datasets analyzed. For the MST measurements, single point measurements were excluded if they were found to have aggregation auto-detected by the instrument.
Replication	For the high-resolution structure obtained in this study, a small dataset was first collected on a 200-kV Talos Arctica electron microscope. The structures were then confirmed and improved by data collection on a 300-kV Titan Krios electron microscope. For the MST measurements, three independent experiments were performed for each construct, and each experiment consisted of three technical replicate scans. Each biochemical reconstitution was performed at least three times and was reproducible. XLMS experiments were performed 1-5 times independently with the same protein preparation for depth coverage and source data are included.
Randomization	Not applicable to this study in general since it did not allocate for experimental groups. In the 3D refinement, particle images were split into two half groups.
Blinding	Blinding is generally not applicable to biochemical studies and structure determination by single-particle cryo-EM and is not used in standard protocols for the experiments described here. Similarly, the MST analysis was not blinded.

Reporting for specific materials, systems and methods

We require information from authors about some types of materials, experimental systems and methods used in many studies. Here, indicate whether each material, system or method listed is relevant to your study. If you are not sure if a list item applies to your research, read the appropriate section before selecting a response.

Materials & experimental systems

n/a	Involved in the study	
☒	☐	Antibodies
☐	☒	Eukaryotic cell lines
☒	☐	Palaeontology and archaeology
☒	☐	Animals and other organisms
☒	☐	Human research participants
☒	☐	Clinical data
☒	☐	Dual use research of concern

Methods

n/a	Involved in the study	
☒	☐	ChIP-seq
☒	☐	Flow cytometry
☒	☐	MRI-based neuroimaging

Eukaryotic cell lines

Policy information about cell lines

Cell line source(s)	Commercial insect cell lines were used for baculovirus production (Gibco Sf9) and protein expression (Expression Systems Tni).
Authentication	All cell lines are commercially available and authenticated by the manufacturers by isozyme and karyotype analysis.
Mycoplasma contamination	Cell lines were tested by the manufacturer for mycoplasma contamination.
Commonly misidentified lines (See ICLAC register)	None.
