# Supplementary material for: Cryo-EM structure of the human CST–Polα/primase complex in a recruitment state
Source: Nat Struct Mol Biol. 2022 May 16;29(8):813–9. doi: 10.1038/s41594-022-00766-y (PMC9371972; doi:10.1038/s41594-022-00766-y)
Supplement: Source Data Extended Data Fig. 1 — Unprocessed Gels [file 41594_2022_766_MOESM5_ESM.pdf]

Cai et al. Source Data (uncropped gels) - Extended Data Fig. 1

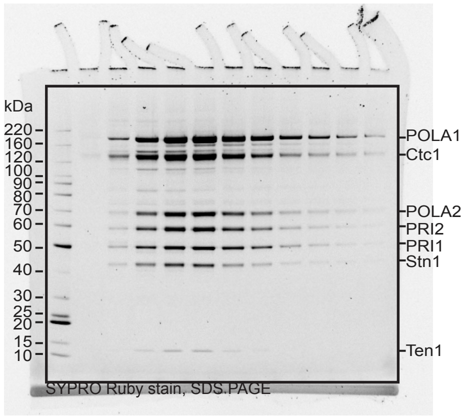

Extended Data Figure 1a

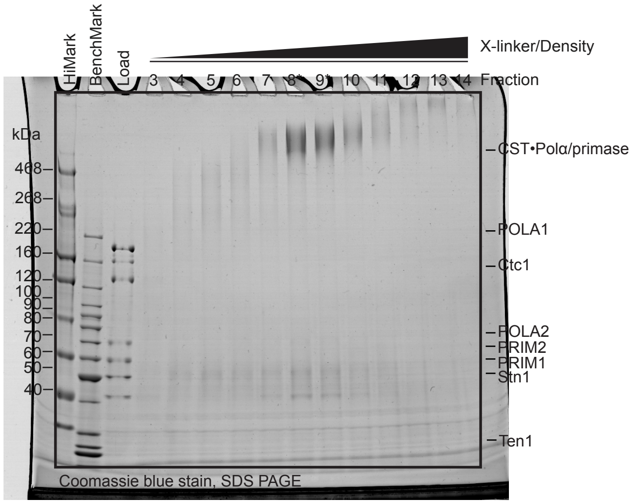

Extended Data Figure 1c

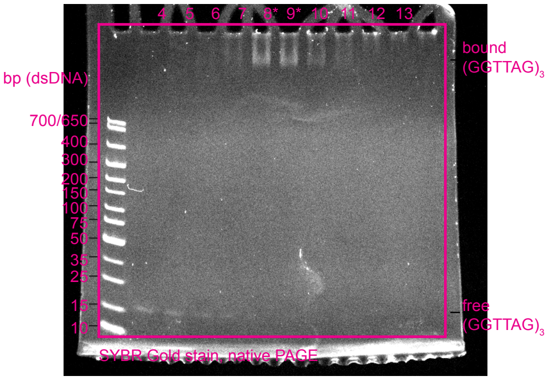

Extended Data Figure 1c
