# Supplementary figures and images for: Cryo-EM structure of the human CST–Polα/primase complex in a recruitment state
Source: Nat Struct Mol Biol. 2022 May 16;29(8):813–9. doi: 10.1038/s41594-022-00766-y (PMC9371972; doi:10.1038/s41594-022-00766-y)

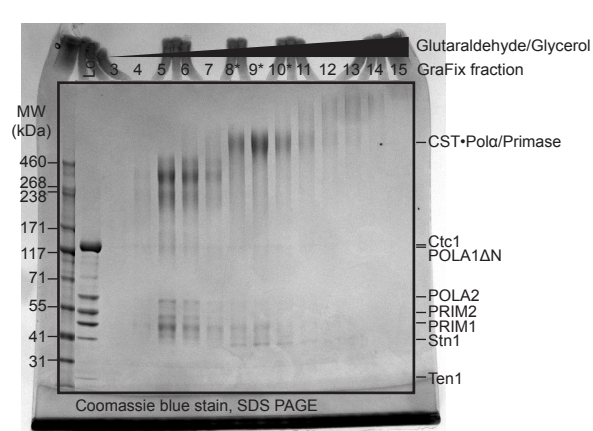

Extended Data Figure 3a

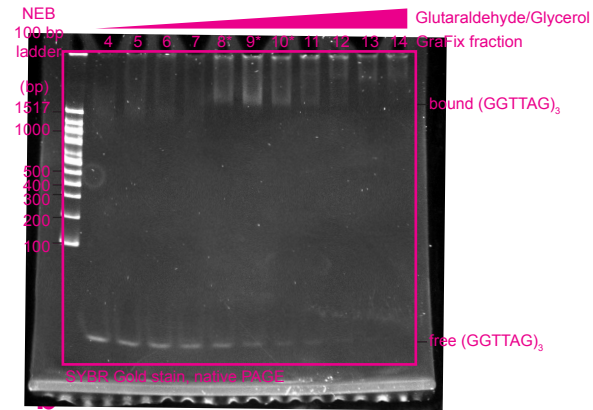

Extended Data Figure 3a

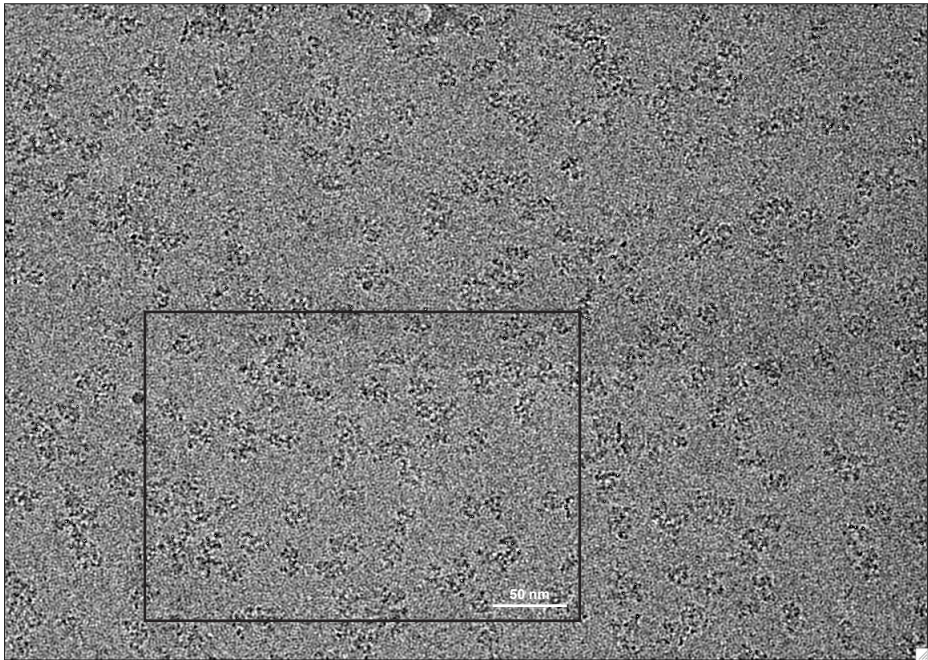

Extended Data Figure 3d

Supplement: Source Data Extended Data Fig. 3 — Unprocessed Gels, Uncropped Micrograph [file 41594_2022_766_MOESM6_ESM.pdf]

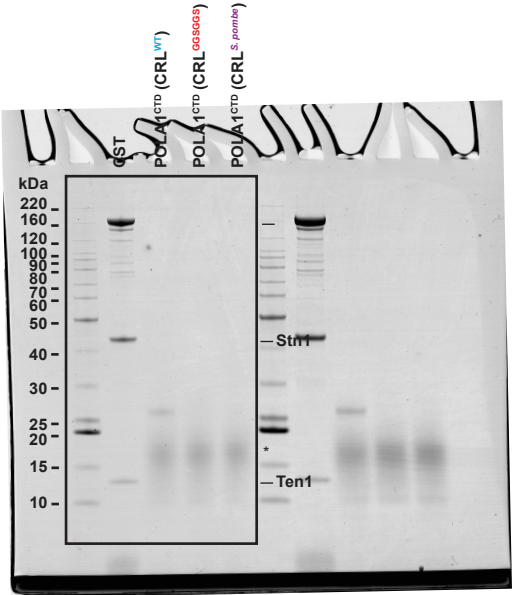

Extended Data Figure 6a

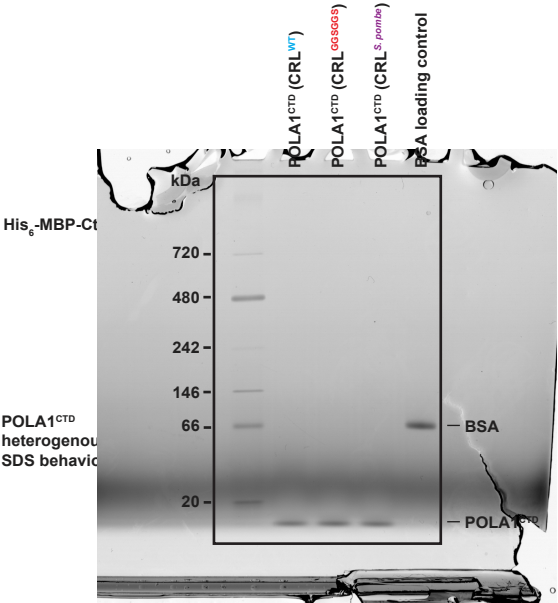

Extended Data Figure 6b

Supplement: Source Data Extended Data Fig. 6 — Unprocessed Gels [file 41594_2022_766_MOESM7_ESM.pdf]

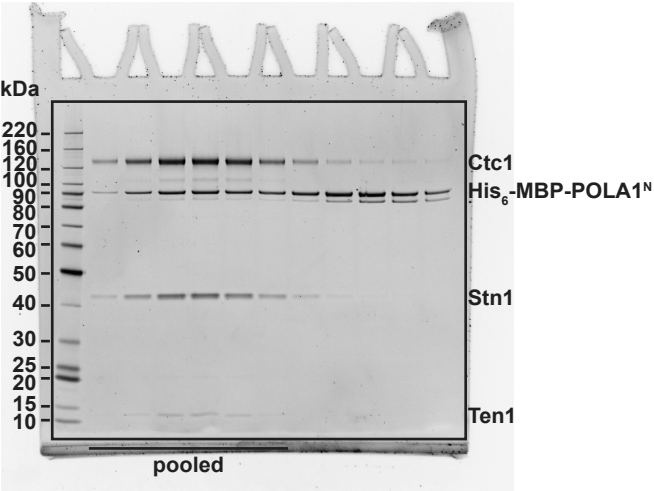

Extended Data Figure 7a

Supplement: Source Data Extended Data Fig. 7 — Unprocessed Gels [file 41594_2022_766_MOESM9_ESM.pdf]
